# A social identity perspective on interoperability in the emergency services: Emergency responders' experiences of multiagency working during the COVID‐19 response in the UK

**DOI:** 10.1111/1468-5973.12443

**Published:** 2022-12-26

**Authors:** Louise Davidson, Holly Carter, Richard Amlôt, John Drury, S. Alexander Haslam, Matthew Radburn, Clifford Stott

**Affiliations:** ^1^ School of Psychology University of Sussex Brighton UK; ^2^ School of Psychology, Behavioural Science and Insights Unit UK Health Security Agency London UK; ^3^ School of Psychology University of Queensland Queensland St Lucia Australia; ^4^ School of Psychology Keele University Staffordshire UK

**Keywords:** COVID‐19, emergency response, interoperability, multiteam systems, social identity

## Abstract

Recent research has shown that multiagency emergency response is beset by a range of challenges, calling for a greater understanding of the way in which these teams work together to improve future multiagency working. Social psychological research shows that a shared identity within a group can improve the way in which that group works together and can facilitate effective outcomes. In the present study, 52 semistructured interviews were conducted with 17 strategic and/or tactical responders during the COVID‐19 pandemic to understand the possible role of shared identity in the multiagency response to COVID‐19 and whether this was linked to factors that facilitated or challenged interoperability. Findings show evidence of a shared identity at a horizontal intergroup level among responders locally. However, there was limited evidence for a shared identity at the vertical intergroup level between local and national responders. Three key factors linked to shared identity appeared to contribute to effective multiagency working. First, pre‐existing relationships with other responders facilitated the ease with which responders were able to work together initially. Second, a sense of ‘common fate’ helped bring responders together, and finally, group leaders were able to strategically reinforce a sense of shared identity within the group.

## 1 INTRODUCTION

Major incidents and emergencies can have devastating effects on both human welfare and society, as demonstrated in recent examples from the United Kingdom (UK)—for example, the Manchester Arena attack and the Grenfell Tower fire (both 2017), the Salisbury nerve‐agent attack (2018), and the COVID‐19 pandemic (2020–present).

When major incidents and emergencies occur in the UK, responders from the three emergency services (Police, Fire and Rescue Service [FRS] and Ambulance) and partner agencies are required to come together as multiagency teams to work collaboratively on the response to achieve the superordinate goal of saving lives and reducing harm (Cabinet Office, 2013). During this kind of response, interoperability between responding agencies is vital. As defined in the Joint Emergency Services Interoperability Principles (JESIP), interoperability is ‘the extent to which organizations can work together coherently as a matter of routine’ (JESIP, 2013, p. 2). However, interoperability has been consistently highlighted as a key challenge that hinders effective joint working. Indeed, in a review of 32 major incidents between 1986 and 2010, coordination between responding agencies was identified as a persistent challenge (Pollock, 2013). To address this, JESIP was introduced to improve interoperability by providing five principles for joint working: colocate, communicate, coordinate, jointly understand risk and shared situational awareness (JESIP, 2021, Table 1).

**Table 1:** The five principles for joint working (JESIP, 2021)

Principle	Description
Colocate	Colocate with commanders as soon as practicably possible at a single, safe and easily identified location near the scene.
Communicate	Communicate using language which is clear and free from technical jargon and abbreviations
Coordinate	Coordinate by agreeing on the lead service. Identify priorities, resources, capabilities and limitations for an effective response, including the timing of further meetings.
Jointly understand risk	Jointly understand risk by sharing information about the likelihood and potential impact of threats and hazards, to agree on appropriate control measures.
Shared Situational Awareness	Shared Situational Awareness was established by using METHANE—an established reporting framework that provides a common structure for responders and their control rooms to share incident information (see JESIP, 2021, p. 16) – and the joint decision model—a model used to bring together available information, reconcile potentially differing priorities and then make effective decisions together (see JESIP, 2021, p. 19).

Abbreviation: JESIP, Joint Emergency Services Interoperability Principles.

Yet, even with the introduction of JESIP, interoperability has continued to be highlighted as a challenging issue in emergency response. For example, Part 2 of the Manchester Arena Inquiry found ‘significant failures in relation to each of [the JESIP] principles for joint working on the night of the Attack’ (Manchester Arena Inquiry Volume 2‐I, 2022, p. 45). For example, communication challenges between the emergency services led to significant delays in the FRS arriving at the scene (see also Kerslake, 2018). However, these are not just UK‐based challenges. Similar challenges have been highlighted in emergency response internationally, not only in Europe (e.g., Sweden: Palm & Ramsell, 2007; Wimelius & Engberg, 2014; and the Netherlands: Bharosa et al., 2009), but also across the globe (e.g., the United States: Majchrzak et al., 2007; National Commission on Terrorist Attacks Upon the United States, 2004; Indonesia: Rencoret et al., 2010 and Haiti: Patrick, 2011). This suggests that lessons identified in previous reports are not being learned (cf. Pollock, 2017, 2021) and highlights the need for a better appreciation of the persistence and intractability of interoperability challenges.

Interoperability has also been foregrounded by the COVID‐19 pandemic. The pandemic has required a multiagency response both at the vertical level, involving interactions between local and national groups (e.g., between local multiagency groups and government agencies), but also at the horizontal level, involving interactions between groups locally (e.g., between different emergency service organizations). Accordingly, in 2020, multiagency coordination groups were established across the UK to bring together local responders from organizations including the emergency services, local authorities, and other key organizations to provide a joined‐up response to COVID‐19.

In the present research, we zero in on the multiagency response to COVID‐19 to try to better understand the factors that might facilitate or challenge interoperability. For this purpose, we carried out a series of semistructured interviews with Police, FRS and Ambulance responders from across the UK who were involved in the COVID‐19 response at a strategic and/or tactical level. Specifically, we sought to address a gap in current research by exploring the role of shared identity in multiagency working in this context: was this relevant, how did it arise and how did it function? However, before describing this research, we first seek to better understand the context in which multiagency working operates by summarizing research on multiteam systems (MTSs), before introducing the Social Identity Approach and its relevance to this research.

### 1.1 Multiteam systems

MTSs are comprised of at least two teams that work directly and interdependently to achieve a collective goal (Mathieu et al., 2001; Shuffler et al., 2015). They centre on the dynamics of subteams nested within a superordinate team. Each team within an MTS possesses specialist skills and may have different individual goals that contribute to the same collective goal (Davison et al., 2012). In contrast to traditional teams, MTSs require team members to coordinate effectively both *within* their individual subteam, as well as *across* the teams that form the superordinate MTS. For example, in multiagency emergency response, the usually separate organizations of the Police, FRS and Ambulance Services form subteams that are nested within the superordinate team of the emergency services. Each of these teams has different subgoals—for example, neutralizing a threat (Police), stabilizing the structure of a building (FRS) and treating casualties (Ambulance)—which all contribute to the collective superordinate goal of saving lives and reducing harm.

MTS research has been applied to high‐risk and dynamic settings such as military operations (e.g., DeCostanza et al., 2014; Liu et al., 2003) and medical emergencies (e.g., Mathieu et al., 2001). More recently, this theoretical perspective has been applied to emergency response (e.g., Brown et al., 2021; Waring et al., 2020). To this end, Waring et al. (2020) conducted naturalistic observations during two large‐scale emergency response exercises to examine the processes used to make joint decisions in MTSs operating in extremis. They found that the effectiveness of decision‐making varied across decision‐making groups. The authors suggested that differences in communication were a key cause of this variability. For example, responders provided agency (subgroup) specific updates to the MTS (superordinate group), and this has been shown to interrupt decision‐making (e.g., Waring et al., 2018). Leaders' relationships with different teams also played a key role in shaping discussions and outcomes.

This research shows that the composition of the group can be an important determinant of effective group decision‐making (see Bang & Frith, 2017, for a review). Adding to this, in a recent review of collaboration and governance in the emergency services, Wankhade and Patnaik (2020) called for a better understanding of the social interactions that take place during multiagency work to understand how collaborations can be better managed (see also Van Scotter & Leonard, 2022). To address this gap, further research is needed into the dynamics of group processes on the ground in these unique contexts. More specifically, to make interoperable working as effective as possible, we need to understand how individuals from separate organizations come to work together interdependently as a group.

As a theoretical framework to guide this exploration, social identity theorizing has been used to help researchers and practitioners understand how groups operate in MTSs. For example, MTSs whose subgroups share a superordinate identity have been found to collaborate more effectively (Mell et al., 2020). Furthermore, Cujipers et al. (2016) conducted a command‐and‐control firefighting computer simulation whereby participants were in a team and had different roles and responsibilities. They found that participants' identification with their MTS was positively associated with MTS performance, but that interteam task and relationship conflicts mediated this relationship. This suggests that in an emergency response situation, identification with the response team can help to improve the effectiveness of the response. Thus, based on the call for a better understanding of the social interactions that take place during a multiagency response, and the clear relevance of social identity to this matter, we are using the Social Identity Approach as a theoretical guide for this research. Below, we introduce the Social Identity Approach and discuss how shared identity might help us better understand interoperability challenges during a multiagency (or MTS) response.

### 1.2 The Social Identity Approach

The Social Identity Approach is a social psychological framework that seeks to understand the distinct contribution that group life makes to people's psychology and behaviour. The approach is comprised of two interrelated theories—*social identity theory* (Tajfel & Turner, 1979) and *self‐categorization theory* (Turner et al., 1987)—that are built upon a foundational insight that as well as defining themselves, and behaving, in terms of their personal identity as individuals (Turner, 1982), people can, and often do, also define themselves and behave, in terms of their *social identity* as members of social groups (Tajfel & Turner, 1979). So, whereas personal identity defines a sense of ‘I’ and ‘me’ that describes a person in contrast to others, social identity defines the self in terms of ‘we’ and ‘us’ in ways that psychologically connect people to other members of their in‐group.

There are several factors that facilitate the development of a shared identity between individuals, including a shared sense of common fate (i.e., the feeling that ‘we're all in this together’; Brewer, 2000; Drury, 2018), and effective identity leadership (i.e., helping group members see themselves as ‘we’ as opposed to ‘I’; Steffens et al., 2014). Importantly, in a range of social and organizational contexts, this sense of social identity is observed to be the primary driver of people's behaviour primarily because, as Turner (1982, p. 21) argues, it is what ‘makes group behaviour possible’ (cf. Haslam et al., 2003). In particular, a shared identity within a group is a basis for coordination and cooperation between group members because it increases their psychological sense of interconnection and common purpose (Haslam et al., 2022, 2009). At the same time, social identity provides group members with a basis for developing a shared understanding of situations, as well as common norms for behaving in those situations (Reicher et al., 2010). Consequently, these shared definitions and common norms can improve group behaviour in those who perceive themselves to share social identity (i.e., who are bound together by a common sense of ‘us’; Drury et al., 2009; Haslam et al., 2009) while also fostering trust and respect among group members (Haslam et al., 2012; Turner et al., 1987).

Demonstrating these positive effects, Haslam et al. (2009) showed that individuals who had high group identification were more willing to display organization citizenship than those with lower levels of identification. More generally, a large body of research demonstrates that when group members perceive themselves to share a social identity, this increases their motivation to contribute to the group's success, as well as their ability to do so (as reviewed by Ellemers et al., 2004; Haslam, 2004).

**Figure 1:**
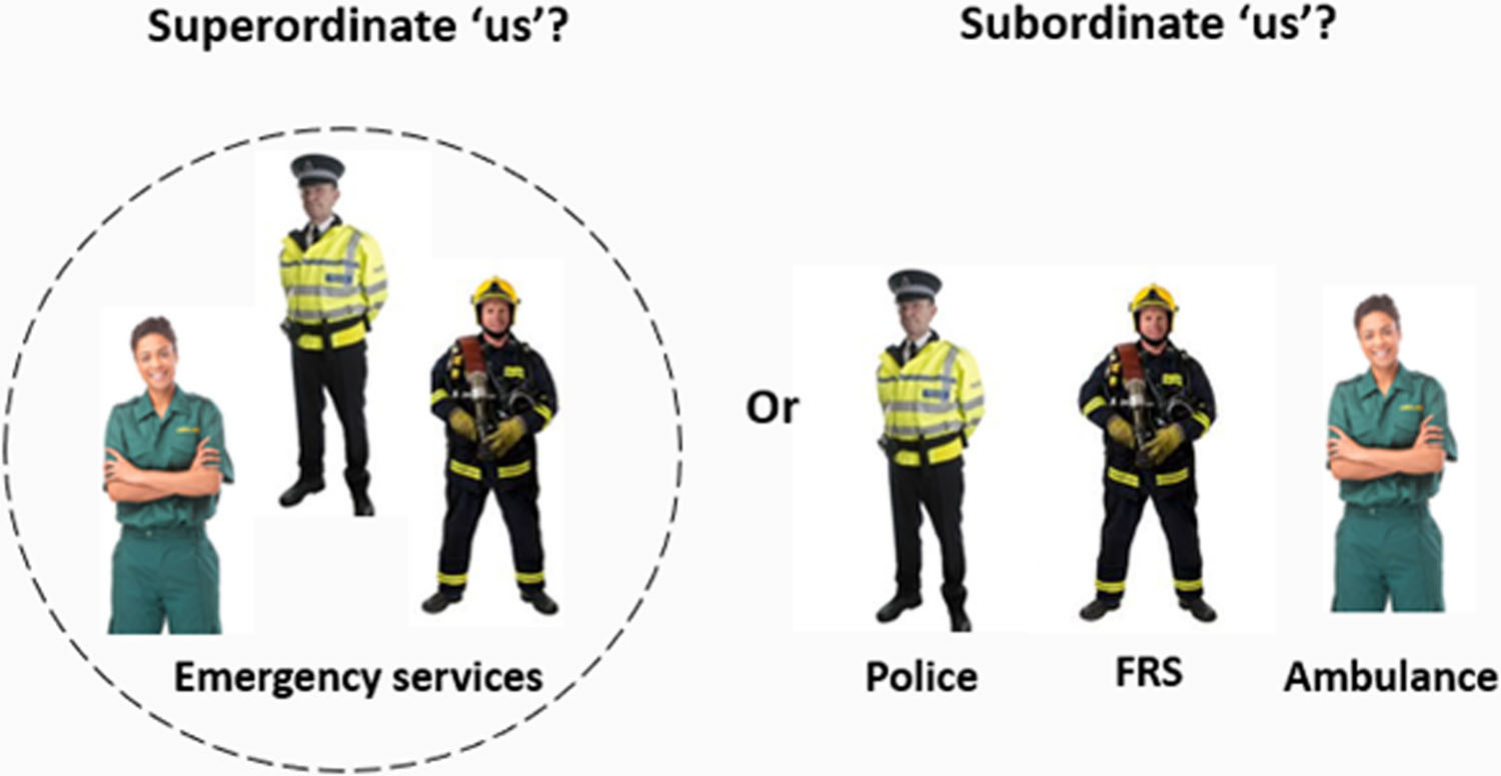
Schematic representation of identities at different levels of abstraction within the emergency services. FRS, Fire and Rescue Service.

At the same time though, people also have multiple social identities which can become salient in different contexts (e.g., us women, us Londoners, us paramedics; Millward & Haslam, 2013; Turner et al., 1987). According to the self‐categorization theory, these can also be defined at multiple *levels of abstraction* (Turner, 1985). For example, a paramedic, Anne, can define herself, as a member of a particular team, as a member of a particular profession, or as an emergency worker (see Figure 1). It follows too that this is likely to have a significant bearing on her behaviour. For example, when (and to the extent that) she defines herself as a member of a particular team, Anne should be motivated to advance the interests of that team; but when (and to the extent that) she defines herself as an emergency worker, Anne should be motivated to advance the interests of emergency workers.

But, *when* do these identities become salient in a given context? When might Anne identify as a paramedic rather than an emergency worker? In this regard, the self‐categorization principles of *fit* and *perceiver readiness* allow us to understand which of many identities might become salient, and therefore guide perception and behaviour, in a given context (Oakes, 1987; see Turner & Reynolds, 2012, for an overview). First, we would expect an in‐group category to become salient when a person perceives the differences between themselves and fellow in‐group members to be smaller than those between in‐group and out‐group members (also known as *comparative* fit; Haslam, 2004). For example, if Anne (a paramedic) was surrounded by paramedics and police officers, we would expect her identity as a paramedic to be salient. At the same time though, the nature of these differences must be consistent with Anne's expectations about the groups (also known as *normative* fit; Haslam, 2004). For example, Anne's identity as a paramedic is less likely to be salient if the paramedics and police officers are seen to be different from each other in ways that don't fit Anne's stereotypes—for example, if the paramedics are only concerned with threat neutralization, something which is usually a Police priority.

Importantly too, the principles of fit work in interaction with *perceiver readiness* (or *accessibility*, Oakes et al., 1994; Turner et al., 1994). This refers to the ways in which our willingness to take on a given social identity is determined by such things as our personal history and our strength of prior identification (Haslam, 2004). For example, if Anne has worked as a paramedic for a long time, and has a strong commitment to her job, but has limited experience working as part of a team with police officers and firefighters, she may be more likely to identify as a paramedic than an emergency worker.

In line with this understanding that people can have multiple identities, it is beneficial to understand which of these identities is most likely to be important in a particular context. For example, is Anne's commitment to the emergency services, and thus her ability to work in a group with police officers and firefighters, likely to be greater if her subgroup identity of being a paramedic is denied?

To address this question, Mühlemann et al. (2022) recently proposed the Social Identity Model of Organizational Change (SIMOC). This suggests that employees will identify with the newly emerging organization and adjust to organizational change more successfully if they are able to maintain their pre‐existing identity. On the other hand, when pre‐existing identities could not be maintained, adjustment to organizational change was determined by the extent to which employee supervisors helped to create and promote a new, positive, and meaningful organizational identity (Mühlemann et al., 2022). Thus, the development of a successful group identity following two groups merging is facilitated when subgroup identities are not denied, but instead, individuals are able to hold onto their subgroup identities. With this in mind, it could be argued that Anne's commitment to being an emergency worker will be greater when her subgroup identity as a paramedic is maintained within the superordinate identity. Alternatively, the newly formed groups will require a leader who helps to create and promote a positive and meaningful identity for the group (see also Haslam et al., 2021; Hogg, 2001; van Knippenberg & Hogg, 2003).

This analysis is clearly relevant to interoperability in multiagency response because the organizational entities here typically involve individuals from usually independent organizations who are required to work collaboratively with each other. In particular, responders are required to jointly provide the overall multiagency management of the incident, yet they still have agency‐specific responsibilities (Cabinet Office, 2013), which can conflict with the overall superordinate goal of the response (Mathieu et al., 2001). As a result, there is a need to understand what factors impact interoperability in a multiagency response to improve the effectiveness of these unique groups in future incidents.

While previous research has examined the role of social identity in an emergency response setting (e.g., Cujipers et al., 2016), the participants in this study were undergraduate students, not emergency responders. Accordingly, while it provides useful insight into the ways in which shared identity might be linked to MTS performance, it does not provide direct insight into the challenges of interoperability experienced by emergency responders on the ground during multiagency response. Doing so is a key goal of the present study.

### 1.3 The present study

Existing research highlights the recurring challenges that arise in multiagency response and calls for a greater understanding of the way in which these teams work together, to improve multiagency working in the future. Potentially exacerbating existing challenges, the COVID‐19 pandemic has presented a unique set of challenges to emergency responders, in terms of the scale, longevity, and complexity of the response required. For example, most emergencies in the UK are handled locally with no direct involvement from a national level (Cabinet Office, 2013). Here, interoperable working requires positive relationships between those at the horizontal (local) level. However, the COVID‐19 response, central Government played a leading role in the response. This added additional considerations for responders to manage this vertical relationship, as well as their horizontal relationships.

With this in mind, we conducted regular, semistructured interviews with responders involved in the COVID‐19 response at a strategic and/or tactical level from across the UK. The purpose of this was to understand the possible role of shared identity in the multiagency response during the initial months of the pandemic in 2020.

More specifically, the aims of this research were to address the following research questions (RQs):

RQ1. Was there any evidence of a shared identity between responders?
RQ2. What factors facilitated or challenged effective multiagency working?
RQ3. If there was a sense of shared identity, was this linked with any of the factors that facilitated or challenged effective multiagency working?

## 2 METHODS

### 2.1 The emergency response context in the UK

Within the UK, the multiagency response to major incidents and emergencies is managed through a three‐tiered command structure: strategic, tactical and operational (as set out in the Civil Contingencies Act, CCA, 2004; see Table 2). This structure is comparable to that in other countries which also adopt a three‐tiered command structure for emergency management. For example, Belgium uses a similarly structured strategic–tactical–operational command system, and Sweden's command is separated into a system‐operational‐task commands (Bram et al., 2016).

**Table 2:** Tiers of command and associated responsibilities emergency responders adopt when responding to incidents (JESIP, 2021)

Tiers of command	Associated responsibilities
Strategic	Sets the strategic direction Coordinates responders Prioritizes resources
Tactical	Interprets the strategic direction Develops the tactical plan Coordinates activities and assets
Operational	Implements the tactical plan Commands the single‐organization response Coordinates actions

Abbreviation: JESIP, Joint Emergency Services Interoperability Principles.

This tiered structure brings together partners from two categories: Category 1 (e.g., the emergency services, local authorities, the National Health Service) who serve a leading role in the response and are involved with most incident responses; and Category 2 (e.g., the Highway Agency and public utility companies) who provide support when incidents affect their sector, and thus the Category 2 responders present varies between incidents (Cabinet Office, 2013).

During a joint response, multiagency coordinating groups are often established at the strategic (Strategic Coordinating Group, SCG) and tactical (Tactical Co‐Ordinating Group, TCG) levels (Cabinet Office, 2013). Here, representatives from relevant agencies come together to provide a vital coordination role in an incident response (CCA, 2004). Thus, interoperability between responders is critical. However, as discussed above, interoperability continues to challenge responders during the multiagency response (e.g., Manchester Arena Inquiry Volume 2‐I, 2022; Moore‐Bick, 2019; Pollock, 2013, 2017, 2021).

### 2.2 Procedure

Fifty‐two semistructured interviews were conducted with 17 responders from Police (*N* = 8), FRS (*N* = 7) and Ambulance Services (*N* = 2) across the UK who were involved in the COVID‐19 response at a strategic and/or tactical level—all responders were involved in the SCG and/or TCG within their local area (see Table 3, for a full list of participant details). Repeated interviews took place between April 13th, 2020 and July 27th, 2020. Potential participants were identified by word of mouth, initiated through pre‐existing contacts with the research team, and recruited for the study via email. Due to commitments in the ongoing COVID‐19 response, responders took part in an unequal number of interviews, ranging between 1 and 12 each (*M* = 4, SD = 3.15). To ensure anonymity, responders were given a unique participant number (1–17; see Table 3).

**Table 3:** Participant information

Participant	Organization	SCG/TCG	Region	Gender	Number of interviews conducted
1	FRS	SCG	Wales	Male	1
2	FRS	SCG	London	Male	12
3	FRS	SCG	London	Male	6
4	Police	TCG	East	Male	3
5	Police	SCG	Wales	Male	9
6	Police	TCG	West Midlands	Male	4
7	Ambulance	SCG	West Midlands	Male	2
8	Police	SCG	Northern Ireland	Male	2
9	FRS	SCG	South East	Male	1
10	FRS	SCG	South East	Male	1
11	FRS	SCG	South East	Male	3
12	Police	TCG	Wales	Male	1
13	FRS	TCG	London	Male	1
14	Police	SCG and TCG	North West	Male	2
15	Police	TCG	South East	Male	2
16	Police	TCG	South East	Female	1
17	Ambulance	SCG and TCG	Scotland	Male	1

*Note*: The participant's age was not recorded.

Abbreviations:FRS, Fire and Rescue Service; SCG, Strategic Coordinating Group; TCG, Tactical Co‐Ordinating Group.

Interviews took place either over the telephone or via the online platform, Microsoft Teams, and were recorded with a dictaphone. Before their first interview, responders were provided with an information sheet electronically. A verbal consent protocol was read out to responders before their first interview, and they were asked to verbally consent to take part.

Subsequent interviews were carried out between 6 and 56 days after the previous interview (*M* = 17 days, SD = 13.2). The first interview for each responder lasted on average 41 min (max = ∼57 min, min = ∼26 min). Subsequent interviews lasted on average 23 min (max = ∼42 min, min = ∼11 min).

The interviewer followed an interview schedule during the interview which was developed following discussions between members of the research team. For the first interview, questions focussed around roles and responsibilities (e.g., ‘What is your current role within the COVID‐19 response?’); multiagency working (e.g., ‘Can you tell me about the range of partners that you are involved with in this response?’); strengths and weaknesses (e.g., ‘Can you tell me about any challenges you have faced?’); adaptation (e.g., ‘Are there any specific areas of improvement that you have recognized in this response?’); and training and guidance (e.g., ‘Is there any specific training or guidance you are following in your response?’). Subsequent interviews focussed on any changes or developments in the response since the previous interview. Specific questions relating to social identity were not asked; this allowed responders to discuss matters that were important to them and allowed any reference to social identity to occur spontaneously without direct prompting from the researcher. The full interview schedules can be found in Supporting Information: Materials 1 and 2.

Ethical approval was independently granted by the UK Health Security Agency's Research and Governance Group on April 6th, 2020 (Reference Number NR0196).

### 2.3 Research context

The role of the SCG in the COVID‐19 response was to take overall responsibility for the response and to establish the strategic framework within which the tactical and operational levels of command could operate. On the other hand, the role of the TCG was to provide a coordinated tactical response to COVID‐19. For example, responders discussed that their role included supporting personal protective equipment deliveries, setting up temporary mortuaries, and ensuring the vulnerable population was adequately cared for. In addition, in most of the areas interviewed the Police chaired the SCG and/or TCG. In one area the FRS chaired the SCG. When Police or FRS was not chair, these meetings were chaired by a representative from the health sector.

A summary of key dates, events, and considerations that their SCG and/or TCG needed to discuss to facilitate the operational response during this period and events provided by participants during the interviews is presented in Table 4.

**Table 4:** Summary of key dates, events and response considerations

Time period	Key date	Key event	Summary of response considerations
March	March 26th, 2020	UK nationwide ‘stay at home’ order	N/A
April 13th– May 14th, 2020	May 13th, 2020	Some ‘stay at home’ restrictions in UK eased	Management and delivery of personal protective equipment (PPE) Mortality planning Planning for a potential easing of ‘stay at home’ restrictions Testing key and critical staff for infection of the virus
May 15th–June 12th, 2020	June 1st, 2020	Groups of six allowed to meet outside in England	Revisiting capabilities previously stood up in the response and looking at what can be removed or stood down (e.g., mortality planning, pandemic multiagency response teams, PPE planning) Preparing for a subsequent wave Managing a return to business as usual
June 13th–July 27th, 2020	June 19th, 2020	UK's alert level lowered from Level 4 (severe risk, high transmission) to 3 (substantial risk, general circulation)	Understanding the impact of mass protests (e.g., Black Lives Matter) on the response to the pandemic and virus transmission Understanding and implementing Test and Trace Understanding and implementing a shift out of the ‘response’ phase
	July 18th, 2020	Local authorities were given the power to enforce local lockdown	

Abbreviation: N/A, not applicable.

### 2.4 Data analysis

Interviews were analysed using thematic analysis—a method for identifying, analysing and reporting themes (patterns) in data (Braun & Clarke, 2006). A semideductive approach was utilized—while there were no predetermined themes, the Social Identity Approach provided researchers with a general sense of reference when formulating the RQs and conducting the analysis (e.g., use of ‘us vs. them’ language). Data familiarization involved the lead author listening to the recordings of all interviews and then transcribing sections of interviews relevant to the multiagency response to COVID‐19. Sections of the interviews where responders discussed other response activities that were not specific to the multiagency response to COVID‐19 (e.g., the Black Lives Matter protests during the Summer of 2020) were not included in the transcription. The lead author then read and reread the transcripts to identify sections that were relevant to the three RQs—for example, any evidence that social identity processes might be present, or any factors that might be facilitating interoperability. From this, initial codes were generated for these sections (e.g., ‘communication outside of the local area’; ‘the importance of understanding the purpose of the response groups’). These codes were then reviewed, and potential themes were identified by the researchers (e.g., ‘cross‐area relationships’ and ‘understanding the roles of partners’). Once themes had been identified, these were reviewed, defined and named by the researchers. Following discussions, themes were separated into two key topic areas based on the RQs (‘evidence of shared identity’ and ‘factors impacting multiagency working’). The research team met on a fortnightly basis throughout the study.

## 3 RESULTS

**Figure 2:**
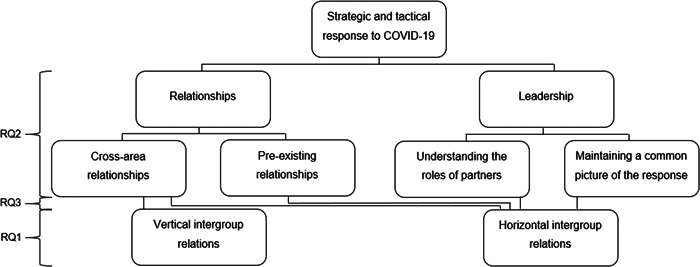
Schematic representation of results related to research questions (RQs): RQ1: Was there any evidence of a shared identity between responders? RQ2: What factors facilitated or challenged effective multiagency working? RQ3: If there was a sense of shared identity, was this linked to any of the factors that facilitated or compromised effective multiagency working?

The results are presented in relation to the two key topic areas derived from the RQs: (i) ‘evidence of shared identity’(RQ1), and (ii) ‘factors impacting multiagency working’ (RQs 2 and 3; see Table 5; Figure 2).

**Table 5:** Overview of topic areas and themes

Topic area	Theme	Definition	Total number of interviews (Interview 1)	Total number of responders	Illustrative quote
Evidence of a shared identity: The extent to which there was evidence of (or lack of) a shared identity in the multiagency group (RQ1).	Horizontal intergroup relations	Responders identifying as part of the collective group (SCG and/or TCG), rather than as their individual organization (Police, FRS or Ambulance).	37 (22)	11	‘I think if anything has come out of this it has shown what we can do if we all convene around a common enemy, whether that enemy in the future is alcoholism, homelessness, obesity, whatever it is, we can convene around it and solve it and I am confident about that, it is a no‐brainer’ (P11, FRS, South East)
	Vertical intergroup relations	The challenging relationship between the national and local levels and a lack of shared identity between the two levels.	29 (11)	12	‘They will only tell you so many hours in advance [it is] very much a top‐down approach which caused complications, which if it had been thought about and not rushed into action like they did […] but what it meant was coordinating and getting the message out to people was really difficult’ (P14, Police, North West)
Factors impacting multiagency working: The mechanisms or pathways that appeared to facilitate or challenge multiagency working (RQ2), and whether responders' shared identity was linked to any of these factors (RQ3).	Relationships: Pre‐existing relationships	Relationships in place between responders before the COVID‐19 pandemic.	19 (12)	14	‘The biggest success is how well everyone has come together in such uncertain terms and put a response in place, and a lot of this is built upon the normal relationships we have across the sectors’ (P4, Police, East)
	Relationships: cross‐area relationships	The relationship between responders from different local areas, either regionally, nationally or internationally.	12 (5)	6	‘There needs to be a mechanism that reminds people to [talk to their neighbouring LRFs] […] This [regional TCG catch up] came about because we know each other […] but this structurally is not written down anywhere, that if you are knee‐deep into an LRF and the whole country is […] it has never happened before, normally we are running an LRF because we are having [bad] weather, not because we are all facing a pandemic, so that is a bit new, so that trigger somewhere in a plan that says if you are running a national incident, speak to your neighbours, speak to your counterparts, and actually have a checklist somewhere of you know, these are the things you want to have a think about’ (P16, Police, South East)
	Leadership: Understanding the roles of partners	Responders have a clear understanding of what the roles and goals of partners from different organizations are in the response to COVID‐19.	16 (10)	12	‘We initially spent a lot of time going through terms and references, and roles and responsibilities, and spent considerable hours going over this in the early stages of the incident. Once they are in and embedded it takes up less time and you get on dealing with the business rather than talking about how the business is going to be structured’ (P4, Police, East)
	Leadership: Maintaining a common picture of the response.	Responders from different organizations all have the same awareness and understanding of the COVID‐19 response.	22 (8)	12	‘That mindset, that message is landing in some areas that “it's over”, you know, stand down the LRF it's over, we're into recovery, happy days. But we're sat here going hang on we are going to be briefed about a second wave and what are we going to do about that, and we have been asked to mitigate for a second wave […] so clearly we are not out of it and not recovering’ (P16, Police, South East)

An illustrative quote is provided for each theme.

Abbreviations: FRS, Fire and Rescue Service; RQ, research question; SCG, Strategic Coordinating Group; TCG, Tactical Co‐Ordinating Group.

Themes are presented alongside representative extracts from the interviews. Responders' unique participant number (1–17), their organization (e.g., Police, FRS or Ambulance) and their region (e.g., South) are presented alongside extracts. Additional extracts can be found in Table 5.

### 3.1 Evidence of shared identity

This topic area relates to the extent to which there was evidence of a shared identity in the multiagency groups. There are two themes under this topic area: ‘horizontal intergroup relations’ and ‘vertical intergroup relations’.

#### 3.1.1 Horizontal intergroup relations

Several responders discussed how they came together as a group to respond to COVID‐19 in terms of an SCG or TCG. Yet, despite coming together physically, there was evidence across the interviews that responders also came together psychologically. For example, a prevalent discussion point during the earlier stages of the response was how the shared threat of COVID‐19 brought responders together. Here, responders used collective terminology when discussing the response. For example, one responder discussed working together for ‘a common purpose’ (P6, Police, West Midlands) and another discussed convening around ‘a common enemy’ (P11, FRS, South East). One responder said that because of the joint threat of COVID‐19, they were able to work collectively together:

We all have this unity, and we are working towards the same goal. (P5, Police, Wales)

Another responder explained how jointly responding to COVID‐19 reinforced the need for multiagency working and the importance of asking for support and sharing information on the skills that they have got between organizations, as well as breaking down any divisions between organizations that were initially present:

We can now have those open conversations between services to address issues as they arise and face challenges as a team as opposed to individual organizations in their own silos. Rather than coming up with individual solutions, what we have come up with now is a combined solution that everyone is comfortable with and that is a testament to the relationships we have got across the organizations and epitomizes the JESIP approach that we are all working together. (P6, Police, West Midlands)

Summing up the idea that all members of the group were part of the same team despite being from separate individual organizations, one responder said they were all doing the same job, regardless of their organizations:

[This is] purely a multi‐agency response that has really worked. If we all take our uniform off, we are working on the same job. (P9, FRS, South East)

One responder recognized that even though members of the group are from different organizations and may view things differently, they are trying to ‘solve the same problem’:

It is those moments where you think you are seeing the world in the same way, but it is about realizing you're not and having the trusted relationships to say it is okay to see the world in that way and it's not wrong, but could we see it in a different way? The idea of a common operating picture, providing a common information set, and we all have the same information and the same problem. (P11, FRS, South East)

#### 3.1.2 Vertical intergroup relations

In contrast to the use of collective language at the local level, when responders were discussing their relationship with national level, responders tended to use ‘*us‐vs‐them*' language, highlighting the disconnect between the two levels:

*They* are doing stuff and *we* are doing stuff. (P15, Police, South East)

This was prevalent when responders were talking about communication between national and local level:

The big unknown locally, which perhaps *they* knew more about nationally, was the degree to which lockdown measures was going to be put in place […] *we* need this information early on. There is a sense that the information exists in Central Government but it's not being shared. (P10, FRS, South East)

Furthermore, responders discussed that the disconnect between the two levels was exacerbated by most communication from the national to local level taking place through media and television announcements:

The big thing with this one is *we* all find out what is going to happen next when *they* stand on the TV and tell *us* what is going to happen next. *We* are finding out at the same time as everyone else, then having to respond to this as a strategic and tactical body. (P4, Police, East)

Discussing what impact this communication had on their ability to respond, one responder said they ‘can't contingency plan’ when they learn about stuff at the same time as the public:

The cat is out of the bag before we have even had a chance to look at it or think about the implications. (P15, Police, South East)

This communication challenge seemed to be exacerbated in the devolved nations of Northern Ireland and Wales. A responder from Northern Ireland said it was challenging to understand whether to follow a UK approach or a devolved approach and often there was mixed messaging between the two administrations which created confusion in the response. Furthermore, a responder from Wales said additional measures had to be put in place before any changes in rules could be enforced leading to further delays in receiving new information. Another responder said this created a ‘false start’ in their response:

[Central Government] constantly fail to say these are the rules for England and everyone just assumes England, Wales and Northern Ireland. Then […] about two hours after the announcement from central government the Welsh government will add a slight twist, by which time the press have got hold of it and people in Wales will read the paper and think oh I am allowed to do that but actually no you're not because the Welsh law is a little bit different […] it [is] difficult trying to enforce the legislation when we have just read the document so aren't fully up to speed with […] *we* are constantly behind the curve. (P5, Police, Wales)

Yet, it was not just *delayed* information sharing between a local and national level which made the response challenging, some responders also said *conflicting* information created challenges. One responder said this caused ‘confidence and reputational issues’ with responders towards Government:

One day *we* are being told to do one thing and the next day something completely opposite comes out. It makes *us* question if *they* really know what they are talking about, why is the change? […] Consideration as to what comes out and even if it is delayed by a day, can avoid a lot of contradiction and confusion. (P6, Police West Midlands)

However, responders in later interviews explained that while the communication challenges were still there later on in the response, they were now able to manage them in a different way. For example, one responder (P15, Police, South East) said once they acknowledged that it was ‘one‐way traffic’ in terms of communication between the national and local levels, they were able to deal with it and manage it better, rather than being disappointed. Another responder (P5, Police, Wales) explained that they became ‘less sensitive’ to what Central Government were saying and that instead of waiting for specific guidance about what to do, they responded on the basis of their general interpretation of the guidance. Similarly, one responder (P11, FRS, South East) said that the relationship between local and national levels became ‘stronger’ because they understood that they were not going to get the information they needed, so found a different way of responding. Yet, despite this development, there still seemed to be a lack of evidence of a shared identity between the two, as shown by the continued use of ‘us‐vs‐them' language:

*We* are now saying [to Government] ‘this is what we are going to do, are we wrong? Is there anything we are missing?’ This is a much more healthier place to be because *we* aren't putting *them* in a corner, but *we* are still seeking *their* view and linking in with the government body that are supposed to advise *us*. *They* can still ignore *us*, but we now have a plan. (P11, FRS, South East)

### 3.2 Factors impacting multiagency working

This topic area relates to the mechanisms or pathways that appeared to facilitate or challenge multiagency working, and whether responders' shared identity was linked to any of these factors. This topic area is separated into two themes: ‘relationships*'* and ‘leadership'.

#### 3.2.1 Relationships

This theme relates to how the relationships and interactions between the different response group impacted group working. This theme is separated into two subthemes: ‘pre‐existing relationships' and ‘cross‐area relationships'.

*Pre‐existing relationships*: In early interviews, nearly all responders said a key strength of the response was how well everyone came together as a group to put a response in place in such uncertain terms and credited pre‐existing relationships between partners from different organizations in facilitating this:

In a long‐playing incident like this you can utilize those relationships you have already got and work quite effectively as opposed to coming together at the point the incident started and develop the relationships from there. So, having those pre‐existing relationships is what has made this response so effective. (P7, Ambulance, West Midlands)

One responder said that they already trusted each other and saw each other as a ‘unit’ (P5, Police, Wales). Furthermore, one responder described their partnership as ‘well‐oiled’ due to the regular recent incidents they had attended, such as the London Bridge and Finsbury Park attacks, and Grenfell Tower fire (P3, FRS, London). Another responder expanded on this saying they have ‘worked as oneone’ for so long (P2, FRS, London). Yet, it is not just about the *presence* of pre‐existing relationships that helped the group come together, some responders suggested the *quality* of that relationship was also important in group working. For example, knowing people on first name bases and having their phone numbers already saved could help resolve challenges quickly. One responder said that friendly relationships can help ‘lighten the mood’ when the pressure on them is high (P12, Police, Wales). In addition, another responder said friendly relationships are beneficial not just for the current response, but also for *future* group working:

The fundamental foundation of what we have been able to do has been the relationships […] we work hard for each other because we like each other and trust each other and know each other's issues and we have a trusting relationship whereby we can have open conversations. We have committed to each other to develop these relationships which will put us in good stead in the future […] I am confident we can resolve an incident because of the relationships that we have. (P11, FRS, South East)

*Cross‐area relationships*: In later interviews, while communication challenges between national and local levels were still prevalent, some responders reported being less reliant on information coming down from a national level to guide their response, instead taking a more local‐level approach. Responders in the South East credited the development of connections with regional partners which facilitated a common regional approach. They said this allowed responders from different areas to compare and discuss what actions were being taken in each region, to share relevant information and to provide a vital coordination role between regions:

We are now solving the same problem but in a different way […] we have focussed on regional colleagues and partners, […] we have agreed a common approach across the South East. We have a workshop on Thursday to compare approaches to the modelling cells, this has enabled us to start comparing. (P11, FRS, South East)

Furthermore, one responder (P2, FRS, London) talked about lesson sharing nationally to enable other areas to learn lessons from London, who in the early months of the pandemic seemed to be worse affected than other areas of the country. In addition, one responder (P17, Ambulance) in Scotland discussed sharing lessons internationally, which came about due to the strong international links they have. As well as providing *practical* support, this cross‐area connection also provided *emotional* support by ‘providing the opportunity to vent and also assurance’ (P16, Police, South East). This was also echoed in Scotland where one responder said they recognized that several lives had been lost and that this caused an emotional strain for responders:

When London were getting hit about three to four weeks ahead of us, I had a number of one‐to‐one strategic meeting calls with other strategic commanders in London about how does this feel. Not the numbers or sterile meeting room environment but the phone a friend item, how's it going? What does it feel like? (P17, Ambulance)

Pre‐existing relationships with responders from other areas were credited as a key driver behind this cross‐area lesson sharing. The responder from Scotland said they depended on their network and relationships that they had built up with others before COVID‐19 (P17, Ambulance). Furthermore, a responder from the South East said that there was nothing in guidance about talking to people from other regions:

[Having a regional TCG catch up] came about because we know each other […] but this structurally is not written down anywhere, [….] speak to your neighbours, speak to your counterparts and actually have a checklist somewhere […] the things you want to have a think about. (P16, Police)

#### 3.2.2 Leadership

This theme refers to the way that the leaders, or chairs, of the multiagency groups, were able to influence group work. This theme is separated into two subthemes: ‘understanding the roles of partners' and ‘maintaining a common picture of the response'.

Understanding the roles of partners: Several responders said that the biggest difference between the COVID‐19 response compared to other incident responses is that it is a health‐led initiative and they have spent a lot of time trying to understand the nature of the health service. Exacerbating this challenge, one responder (P4, Police, East) said they had a health chair of the SCG who had not previously chaired an SCG before and it took them a couple of weeks to fully understand the purpose of the group. In addition, one responder (P12, Police, Wales) said that the COVID‐19 response involved a number of guest agencies that would not normally be involved in a response, such as a prison and probation service. This created challenges with new partners being initially hesitant to share problems they were facing leading to delays in resolving them. Yet, in later interviews some responders said that this challenge eased as time went by:

That [misunderstanding of roles] was […] across all partners not fully understanding what others can do. As time went on it became clearer what everyone was bringing to the table. (P14, Police, North West)

Further, in an initial interview, one responder (P4, Police, East) highlighted the important role of the chair of the group in facilitating an understanding of roles amongst partners by going over the roles and responsibilities of each partner at the beginning of the response. Later on, another responder said that once they had overcome initial differences between partners, they were able to collectively deal with any challenges, rather than working independently of each other:

[We] now can have those open conversations between services to address issues as they arise and face challenges as a team as opposed to individual organizations in their own silos. Rather than coming up with individual solutions, what we have come up with now is a combined solution that everyone is comfortable with. (P6, Police, West Midlands)

Maintaining a common picture of the response: In later interviews, some responders said that different organizations developed a different understanding of where they were at in the response and that this reduced the shared sense of common fate. One responder who initially talked about the unity of the group said that later in the response the group cohesiveness that was originally formed started to weaken because there was no longer a clear common purpose for why they were convening:

It takes an external threat for everyone to come together to work for the greater good and taking one for the team […] but as soon as that external threat slightly dissipates, even if it is just that we are over the initial peak […] everyone starts petty squabbling and it just unravels from the top. (P5, Police, Wales)

To try to overcome this challenge and maintain group cohesiveness, this responder said they laid out eight strategic goals for the SCG at the beginning of each meeting. Further, another responder said that they began each of their SCG meetings with an overview of the common picture of the incident so that each partner knew exactly what was happening, what the challenges were, and what actions needed to be taken:

At the start of the meeting, you start with this is where we are and these are the previously identified risks […] everyone needs to leave the room with a clear line of sight of everyone else's position […] no matter where they are from, they have a clear line of sight of what is happening […] where the pinch points are and what mitigating action needs to be taken. (P12, Police, Wales)

In response to the changing situation, one responder said that their SCG introduced a new phase called ‘stabilization’ which occurred after the initial response phase, but before the recovery phase. Within this phase, the SCG members were not meeting regularly as they had done in the initial response phase, but partners were still working together and ready to meet again if or when it was necessary. This was so that partners were aware they still had access to the resources and support the SCG could provide:

The other alternative is to close [the SCG] down and the message that sends is message complete [their response to COVID‐19 is over] […] that sends all kinds of dangerous signals so the other alternative is to leave it running in the background so it is technically in existence but there is nothing happening in it […] the SCG is a leadership group of senior people across the partnership saying this is still very important to us. If we all walk away […] what we're saying is it's not very important anymore. (P11, FRS, South East)

## 4 DISCUSSION

In this study, we were interested in exploring the relevance of a social identity perspective on interoperability in the emergency services through presenting evidence from interviews with Police, FRS and Ambulance responders from across the UK who were involved in the COVID‐19 response at a strategic or tactical level. We wanted to understand: (a) whether there was any evidence of a shared identity between responders involved in the SCG and TCG's (RQ1), (b) what factors facilitated or challenged effective multiagency working (RQ2) and (c) whether a sense of shared identity was linked to any of these factors (RQ3). Key challenges responders faced in the response are discussed below alongside potential solutions and how shared identity might be linked to this. Discussion is separated by three potential solutions: *relationships, common fate* and *leadership*.

### 4.1 Key challenges, potential solutions and evidence of a shared identity in the multiagency groups

#### 4.1.1 Relationships

In earlier interviews, pre‐existing relationships were credited by responders as being a key facilitators in initially bringing the multiagency groups together. For example, responders in London talked about several recent incidents where they had responded together. Based on these recently shared experiences, responders in this area said that relationships with responders from other agencies were already established. It is well documented within the social identity literature (Drury et al., 2009; Haslam et al., 2009), and recently in the MTS literature (Cujipers et al., 2016; Mell et al., 2020), that individuals identifying with members of their group can help group working and can foster trust in other group members (Turner et al., 1987). In other words, when these relationships are already formed and individuals have had recently shared experiences with each other, it makes it easier for them to act as a group in the present, which is evidenced in the current research. Despite this, a key challenge several responders highlighted throughout the course of the interviews was that delayed or conflicting communication from a national to local level created difficulties for them in preparing for and providing a timely response. This seemed to be exacerbated in the devolved nations of Northern Ireland and Wales where at times it was unclear whether to follow a national or a devolved approach. Yet, in later interviews, some responders discussed sharing lessons regionally (e.g., responders in the South East discussed their response with other responders in the region), nationally (e.g., responders in London discussed their response with responders from other regions across the UK) and between‐nations (e.g., responders in Scotland discussed their response with responders in other countries) to try to manage this challenge. Thus, it appears some responders utilized relationships with partners outside of their local area as a potential solution to a challenging relationship with national partners. In addition, some responders commented that this cross‐area lesson sharing was facilitated by pre‐existing relationships with responders from different areas. This suggests that these pre‐existing relationships facilitated group behaviour both early in the response and in later stages.

#### 4.1.2 Common fate

Several responders used collective terminology when describing the response, particularly in early interviews when discussing what facilitated group working (e.g., ‘common enemy’, ‘unity’), as discussed under ‘Horizontal Intergroup Relations’. Responders spoke about how the shared threat of COVID‐19 helped to bring them together and gave them a common purpose in the pandemic response. This contrasts with the ‘us vs. them’ language used by responders when describing their relationships with the national level, as discussed under ‘Vertical Intergroup Relations’. As such, it seems that the shared threat of COVID‐19 facilitated the group coming together at a local (or horizontal level), or in other words, it appears the responders experienced a sense of *common fate* during their joint response to COVID‐19. Research shows that a sense of common fate between individuals can facilitate a shared identity between members (e.g., Drury, 2018). Subsequently, this shared identity can encourage helpful and empathetic behaviour between group members (e.g., Levine et al., 2005), enhance people's trust with group members (Cruwys et al., 2020) and increase their willingness to cooperate in working towards group goals (Haslam, 2004). Taken in the context of the current research, the shared threat of COVID‐19 is likely to have contributed to the development of a sense of common fate among responders (evidenced through the use of collective languages, such as referring to the virus as a ‘common enemy’). Thus, this sense of common fate is likely to have facilitated a sense of shared identity and subsequently increased their ability to work together collaboratively on the response.

#### 4.1.3 Leadership

An early challenge discussed by responders in initial interviews was the wide range of partners involved in the response. This included partners who would not typically be involved in incident response, and with whom pre‐existing relationships were not present. Yet, some responders discussed how the Chair of their meetings went over roles and responsibilities at the beginning of the response, or when new partners joined, and how this facilitated their ability to work interdependently with, as opposed to independently, each other. Furthermore, in later interviews when the initial wave of COVID‐19 came to an end, some responders said that the initial sense of shared purpose that was present at the beginning of the response seemed to have waned. As such, some responders said their chair spent time going over where they were at with the response and outlined any outstanding issues to help maintain a common picture of the response amongst responders. Thus, according to some responders', their chair attempted to strategically maintain a shared awareness of the situation in this way and helped facilitate group cohesiveness by ensuring common goals were communicated to all. According to Zehnder et al. (2017), effective leadership can help organizations foster a sense of shared identity among members, in turn, facilitating collaboration between group members (Ellemers et al., 2004) and making the group more likely to succeed in their goals (Carton et al., 2014). Recently, Fladerer, Haslam et al. (2021) showed that leaders were able to reinforce a sense of shared identity amongst group members by using collective language such as ‘we’ as opposed to ‘I’. In turn, this reinforced sense of shared identity within the group was subsequently associated with improved organizational performance, emphasizing the importance of effective leadership. This is also in line with SIMOC which emphasizes the importance of group leaders in helping employees adjust to organizational change by helping to create a new, positive and meaningful identity (Mühlemann et al., 2022). Further, in the context of emergency response, recent research has shown that individual characteristics of the Chair of SCG groups can influence decision‐making processes within the group (e.g., Waring et al., 2020; Wilkinson et al., 2019). For example, in video footage analysis of groups responding to either a simulated major incident or large‐scale exercise, Wilkinson et al. (2019) found between‐group differences in the way decision‐making activities were carried out. The authors suggested a potential reason for these between‐group differences could be due to differences in the composition and characteristics of the group, or the disposition of the Chair. The research in the current paper expands on this evidence base by showing that some responders perceived their group leaders to play a particularly important role in facilitating multiagency working when pre‐existing relationships were not already present, or when the sense of common fate began reducing. This echoes further recent research by Fladerer, Kugler et al. (2021) who found that identity leadership was particularly relevant in situations where coworkers' group identification was low. Thus, this highlights the importance of effective leadership, particularly in new or less well‐established groups, such as emergency response groups which often do not work together on a regular basis.

### 4.2 Strengths and limitations

One limitation of the research presented is that only responders from the blue‐light services were included but the COVID‐19 response involved responders from several different organizations. Because of this, it is difficult to discern whether the challenges discussed by responders were common across responders from other organizations involved in the response. The varying availability of responders may have also biased the results to those who took part in the most interviews. Furthermore, the longitudinal data collection method used in the present study is useful for allowing us to understand how aspects of the response, as well as a shared identity within the response groups, changed over time. Thereby, this provides valuable insight into how these processes develop and change, why this might happen, and what effect this subsequently has on multiagency working. However, a key limitation of this methodology is that due to differences in availability for interviews in the ongoing pandemic response, responders took part in an unequal number of interviews. Because of this, changes over time that are captured in the longitudinal data set are likely to over‐represent those who took part in the most interviews. Yet, despite these limitations, several findings from the current research are echoed in recent research by Hill and colleagues who conducted three reviews of the COVID‐19 response to understand the experiences of local and national strategic decision‐makers (Hill, Guest, Hopkinson, et al., 2020; Hill, Guest, Pickford, Hopkinson, Daszkiewicz, Whitton, Reed, Thomas, et al., 2020; Hill, Guest, Pickford, Hopkinson, Daszkiewicz, Whitton, Reed, & Towler, 2020; see Hill et al., 2021, for a summary). These researchers observed that factors such as collaborative working was facilitated by pre‐existing relationships between partners but were hindered by partners who had no prior knowledge of the structures or procedures of the SCG and TCG groups. This suggests that the experiences of participants in the present research are likely to be both common and generalizable to wider response partners. Furthermore, in a longitudinal case study analysis of one local area response to COVID‐19, Radburn et al. (2022) similarly pointed to the importance of leadership in the local‐level response, as well as the challenges presented by the intergroup relationships between the local and national levels. Of course, it is possible that there are factors other than social identity processes that may have impacted multiagency working in the COVID‐19 response. For example, responders from the South East said they introduced a period of stabilization in between the usual ‘Response’ and ‘Recovery’ phase—an interim control stage to mitigate the risk of secondary impacts occurring, as well as allowing multiagency coordination groups to retain their overall focus on reducing the risk of the current threat (Deeming & Burgess, 2017; cf. Deeming, 2020). Recent research looking at the multiagency response to a simulated terrorist incident also found benefits of a three‐phased approach (Brown et al., 2021). Brown et al. (2021) suggest that an additional phase between response and recovery can increase opportunities for collaborative working across agencies and reduce demands on a single team. As such, it should not be ignored that factors other than social identity processes can also facilitate effective multiagency working. It is also important to acknowledge that the current study was focused on the strategic and tactical response to COVID‐19 which has presented unique challenges for emergency responders in terms of the scale and complexity of the response required. However, because of this, it is unclear whether the findings could generalize to other multiagency responses, and it would be beneficial for future research to examine social identity processes in relation to other types of incidents to allow recommendations to be as useful and transferrable as possible. Finally, while the present study provides useful and valuable insight into how shared identity processes might operate in multiagency response teams, the interview questions did not engage specifically with social identity processes. So, while a non‐directive interview protocol was chosen to allow the interviewees to speak directly about things that were most important to them and to allow discussions of relevant social identity processes to occur spontaneously, important aspects relating specifically to social identity may have been missed following this approach. Furthermore, this study does not provide objective evidence that shared identity is associated with improved interoperability. As such, future research would benefit from exploring this issue further using additional data collection methods, including observations of performance and measures of social identification.

## 5 CONCLUSION

The interviews conducted with strategic and tactical responders involved in the COVID‐19 response provide evidence of shared identity between responders at the local level. This identity was created and initially made salient by responders sharing pre‐existing relationships with each other, which in turn facilitated the way that they were able to work together early in the response. Furthermore, a sense of shared common fate between responders at the local level helped make their shared identity salient early in the response. However, when pre‐existing relationships were not present, or when the initial threat of COVID‐19 began to reduce, Chairs of the multiagency groups played an important role in helping to create or maintain a shared identity—for example, by highlighting the roles and responsibilities of partners or emphasizing the shared goals of the response. On the other hand, however, there was limited evidence of a shared identity at the intergroup level between national and local teams, as evidenced by a strong use of ‘us vs. them’ language on the part of local responders. While in some ways this challenged the local‐level response to COVID‐19, it also helped make salient their shared identity at a local level. Relationships across areas helped to make responders' shared identity more inclusive and facilitated the local‐level response while also providing an outlet for responders at the local level to help them overcome the challenging relationship at the national level.

## 6 PRACTITIONER POINTS

-  Relationships between responders from different organizations should be nurtured to ensure that a shared identity is maintained between responders to facilitate the ease at which they are able to come together for future incident responses. For example, organizations should prioritize multiagency training to allow for relationships between responders to develop in advance of a real incident.
- When responders share difficult or challenging experiences with each other, this can help them feel connected to each other, regardless of their organization. During a joint response, responders should be encouraged to use collective terminology, such as ‘we’ and ‘us’ to facilitate this. Furthermore, multiagency debriefs should be encouraged as this provides a valuable opportunity for responders to meet and discuss their shared experiences with each other.
- Leadership is important in facilitating a shared identity and chairs of the multiagency groups can help strategically embed a shared identity if relationships are not already present or if there is not a strong sense of common fate between responders. Chairs can achieve this through specific actions such as making the roles and responsibilities of partners clear and emphasizing shared goals.
- During a nationwide response, strategic and tactical responders at the local level should be encouraged to talk to their neighbouring areas about the response. This can help us to to create a more inclusive sense of shared identity between responders, as well as provide a useful mechanism for sharing best practices to an ongoing response.

## CONFLICT OF INTEREST

The authors declare no conflict of interest.

## Supporting information

Supporting information.

Supporting information.

## Data Availability

The data that support the findings of this study are available on request from the corresponding author. The data are not publicly available due to privacy or ethical restrictions.
