# Supplementary material for: A social identity perspective on interoperability in the emergency services: Emergency responders' experiences of multiagency working during the COVID‐19 response in the UK
Source: J Conting Crisis Manag. 2022 Dec 26:10.1111/1468-5973.12443. Online ahead of print. doi: 10.1111/1468-5973.12443 (PMC9880684; doi:10.1111/1468-5973.12443)
Supplement: Supplementary file 1 — Supporting information. [file JCCM-9999-0-s001.docx]

**Supplementary Materials 1. First Interview Guide**

Roles and responsibilities

1. How are you coping with the COVID-19 response?

Prompts:

- What is your current role within the response?

- Can you explain the responsibilities associated with that role?

- How does the response to this incident compare to other Civil Contingency emergencies that you have been involved with?

Multi-agency working

2. Can you tell me how your local resilience forum is being managed?

Prompts:

- Who is currently chairing the LRF?

3. Can you tell me about the range of partners that you are involved with in this response?

Prompts:

- Have you had any previous involvement with these partners prior to this incident response?

- Do you understand the roles and goals of the partners you are working with?

Strengths and weaknesses of response

4. How have things gone since the last interview?

Prompts:

- Drawing on specific examples if you can, can you tell me how things have gone well since the last interview?

- Can you tell me about any challenges you have faced?

- Did you overcome these challenges?

Adaptation

5. Are there any specific areas of improvement that you have recognised in this response?

Prompts:

- If so, how have these affected the response to COVID-19?

- If there anything you think could be done to further improve the way this response is being managed?

Training and guidance

6. Is there any specific training or guidance you are following in your response?

Prompts:

- Have you received any updated guidance since the incident has started?

- Do you find that the training you have received has been useful to you in this response?

- Are you aware of the JESIP guidelines? If so, have these influenced the way you have responded to this incident?

Other

7. Is there anything else that you would like to comment on that we haven’t already discussed?
