# Supplementary material for: A social identity perspective on interoperability in the emergency services: Emergency responders' experiences of multiagency working during the COVID‐19 response in the UK
Source: J Conting Crisis Manag. 2022 Dec 26:10.1111/1468-5973.12443. Online ahead of print. doi: 10.1111/1468-5973.12443 (PMC9880684; doi:10.1111/1468-5973.12443)
Supplement: Supplementary file 2 — Supporting information. [file JCCM-9999-0-s002.docx]

**Supplementary Materials 2. Subsequent Interview Guide**

1. How have things gone since the last time we spoke last week?

- Using specific examples if you can, can you tell me about any particular challenges that you have faced since we last spoke?

- Using specific examples if you can, can you tell me about anything that has gone particularly well since we last spoke?

2. [Follow up from previous interview]

3. Is there anything else that you would like to comment on that we haven’t already discussed?
